# Dysregulation of the miR-34a-SIRT1 axis inhibits breast cancer stemness

**DOI:** 10.18632/oncotarget.3394

**Published:** 2015-03-18

**Authors:** Wei Ma, Gary Guishan Xiao, Jun Mao, Ying Lu, Bo Song, Lihui Wang, Shujun Fan, Panhong Fan, Zhenhuan Hou, Jiazhi Li, Xiaotang Yu, Bo Wang, Huan Wang, Honghai Wang, Fei Xu, Yan Li, Qiang Liu, Lianhong Li

**Affiliations:** ^1^ Department of Pathology, Dalian Medical University, Dalian 116044, China; ^2^ School of Pharmaceutical Sciences, Dalian University of Technology, Dalian 116024, China; ^3^ The Key Laboratory of Tumor Stem Cell Research of Liaoning Province, Dalian Medical University, Dalian 116044, China; ^4^ Genomics and Functional Proteomics Laboratories, Departments of Medicine and Medical Microbiology and Immunology, Creighton University Medical Center, NE 68131, USA; ^5^ Institute of Cancer Stem Cell, Dalian Medical University, Dalian 116044, China; ^6^ Department of Human Anatomy, Dalian Medical University, Dalian 116044, China

**Keywords:** miR-34α, SIRT1, CD44+/CD24−BCSCs, stemness

## Abstract

Enforced expression of miR-34a eliminates cancer stem cells in some malignant tumors. Sirtuin-1 (SIRT1) is a direct target of miR-34a. Here we found low levels of miR-34a and high levels of SIRT1 in CD44+/CD24− breast cancer stem cells (BCSCs). MiR-34a overexpression and knockdown of SIRT1 decreased proportion of BSCSs and mammosphere formation. Expression of CSC markers, ALDH1, BMI1 and Nanog was decreased. In nude mice xenografts, stable expression of miR-34a and silencing of SIRT1 reduced tumor burden. Taken together, our results demonstrated that miR-34a inhibits proliferative potential of BCSCs *in vitro* and *in vivo*, at least partially by downregulating SIRT1. The miR-34a-SIRT1 axis may play role in self-renewal of BCSCs.

## INTRODUCTION

Breast cancer is the most common cancer with high morbidity and mortality, and the leading cause of cancer death in females worldwide [1]. Although prognosis of breast cancer has been significantly improved last decades by many effective adjuvant therapies, recurrence and relapse remain still major challenge in the treatment of this disease. Growing evidence suggests that its recurrence and/or relapse may be initiated and maintained by remaining cancer stem cells (CSCs) from either residual tumors or those with intrinsic resistance to adjuvant therapy [2–4]. Relatively, CSCs are a group of rare cancer cells with stem cell properties, such as self-renewal and great potentials of proliferation and differentiation, which are functionally responsible for the initiation and the propagation of tumors [5]. Breast cancer stem cells (BCSCs) with cell surface phenotype of CD44+/CD24*^−/low/Lineage−low^* were first identified and isolated by Al-Hajjet al. in 2003 [6]. BCSCs used as therapeutic targets are now getting more attention to the community of breast cancer treatment [7–10].

MicroRNAs (miRNAs) play pivotal roles in a number of biological processes, including apoptosis, self-renewal, differentiation, and division of cells [11–14]. It has now gained more attention that miRNAs play important role in tumorigenesis and metastasis, and can be functioned as either oncogenes or tumor suppressors [12, 15, 16]. A number of studies have shown that miRNAs, such as miR-34, miR-125, miR-200, miR-205, miR-328, and miR-30, were down-regulated and acted as tumor suppressors in breast cancer [16–22]. The miR-34 family, including miR-34a, miR-34b/c, plays an important role in the p53 networking [17, 23–25]. MiR-34a is always down-regulated due to either an aberrant CpG methylation of its promoter or deletion and/or mutation of p53 in cancer [23, 26–28]. Over-expression of miR-34a induces cell apoptosis, cell cycle arrest, and senescence, leading to suppression of proliferation, invasion, and migration of breast cancer [21, 23, 29, 30]. It becomes much clear that miR-34a works as a tumor suppressor by targeting several oncogenes. Furthermore, miR-34a mimic has become the first microRNA to reach phase I clinical trials [17]. Recent studies revealed that miR-34a plays a vital role in inhibiting CSCs of prostate, pancreatic, and colorectal cancers [25, 28, 31, 32]. MiR-34a could represse c-kit to reduce chemoresistance, migration and stemness of colorectal cancer [25]. MiR-34a inhibits self-renewal and metastasis of the prostate CSCs by repressing CD44 [28]. It inhibits the pancreatic CSCs by targeting bcl-2 and Notch [31].

Sirtuin-1(SIRT1), an NAD^+^-dependent histone deacetylase, potentially regulates the acetylation of transcription factor p53 [33–35]. SIRT1 has been implicated in the maintenance of pluripotency in various types of stem cells [36–39]. Interestingly, SIRT1 has also been found to regulate the growth and survival of leukemia stem cells (LSCs). Li *et al*. [40] reported that SIRT1 down-regulation improved the acetylation and subsequent transcriptional activity of p53 in CML progenitors, thus enhanced elimination of CML stem cells in combination with imatinib. SIRT1 plays a critical role in multiple aspects of cancer drug resistance, and promotes the survival of CSCs [41]. SIRT1 has been confirmed to be the direct target of miR-34a [30], and miR-34a, SIRT1 and p53 can form a regulatory feedback loop [42]. Activation of p53 increased the miR-34a expression while miR-34a targeted SIRT1, then down-regulation of SIRT1 stimulated the acetylation and increased the activity of p53 [42], and led to a p53-dependent apoptosis. However, the roles of miR-34a and SIRT1 in the self-renewal of BCSCs are still unknown.

In this study, we found a reverse relationship between the endogenous expressions of miR-34a and SIRT1 in CD44+/CD24^−^ BCSCs. Overexpression of miR-34a reduced cell proliferation rate and induced apoptosis in MCF-7 cells, similarly with the effects of small-interfering RNA against SIRT1. More importantly, either an ectopic expression of miR-34a or knock-down of SIRT1 exhibited a decreased proportion and mammosphere formation capacity of BCSCs. Consistently, BCSCs surface marker ALDH1 [43], self-renewal associated gene BMI1 [20], and stem cell marker Nanog [44] were downregulated at protein levels. Our *in vivo* study further showed that subcutaneous injection of nude mice with either miR-34a overexpressing or SIRT1 knocking down MCF-7 cells resulted in smaller tumors than injection of control cells. The immunohistochemistry showed that ALDH1 was inhibited correspondingly. These results suggest that miR-34a might have a critical role in the self-renewal of BSCSs, and this effect is achieved possibly through down-regulating SIRT1.

## RESULTS

### Endogenous expression of miR-34a and SIRT1 in CD44^+^/CD24^−^ BCSCs

**Figure 1:**
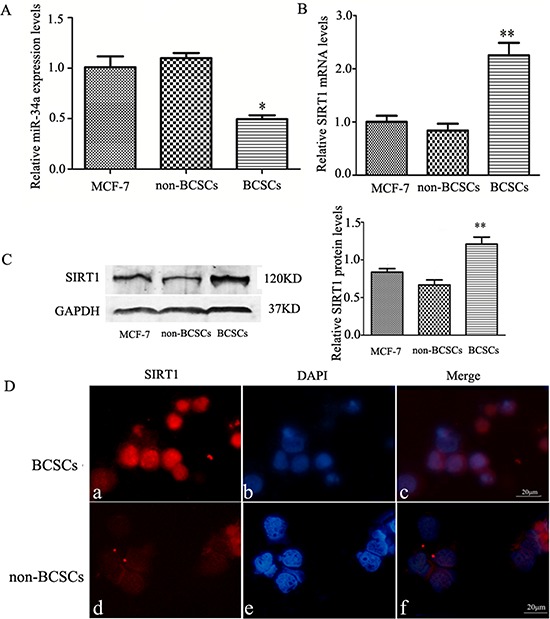
The endogenous expression levels of miR-34a and SIRT1 in CD44+/CD24−BCSCs **A.** Relative qRT-PCR quantification of miR-34a in MCF-7 cells, non-BCSCs, and CD44^+^/CD24^−^ BCSCs. The relative expression levels (mean ± S.D) were normalized to RNU6B, and the expression of miR-34a in MCF-7cells set at one. Compared to MCF-7 cells and non-BCSCs, miR-34a in BCSCs showed significantly lower expression. *p < 0.05. **B.** Representative mRNA expression of SIRT1 in MCF-7(unseparated) cells, non-BCSCs, and BCSCs. The realtive expression levels are presented by setting mRNA levels in MCF-7 cells as one. GAPDH was used for normalization. Expression of SIRT1 mRNA in BCSCs showed > 2 fold higher compared to MCF-7 cells or non-BCSCs. **p < 0.01. **C.** The protein expression of SIRT1 in MCF-7 (unseparated) cells, non-BCSCs, and BCSCs was determined by Western blotting (*left*). The quantitative results were analyzed by Gel-Pro Analyzer 4.0 software (*right*), and GAPDH was used as an endogenous control. **p < 0.01. **D.** Immunofluorescence staining of SIRT1 in MCF-7 cells. a, d, PE-labeled anti-SIRT1(red). b, e, labeled with DAPI (a nuclear marker) (blue). c, f, represent overlay image of a and b, d and e, respectively. SIRT1 were highly expressed in BCSCs than non-BCSCs. scale bar = 20 μm. MCF-7 represents the cells before sorting, non-BCSCs represents non-CD44+/CD24− breast cancer cells, and BCSCs represents CD44^+^/CD24^−^ breast cancer stem cells.

Levels of the endogenous expression of the miR-34a and the SIRT1 in CD44^+^/CD24^−^ BCSCs was estimated by using relative qRT-PCR. We found a lower expression level of miR-34a (Figure 1A), and remarkably higher mRNA level of SIRT1 in BCSCs (Figure 1B). The SIRT1 protein expression level further confirmed by western blot in CD44+/CD24^−^ BCSCs (Figure 1C), which was also verified by immunofluorescence analysis (Figure 1D). Our results from Figure 1 showed an inverse relationship between miR-34a and SIRT1 in CD44+/CD24^−^ BCSCs, suggesting that SIRT1 may be a target of miR-34a in BCSCs.

### Inhibitory effect of miR-34a-SIRT1 axis on cell proliferative potential in MCF-7 cells

**Figure 2:**
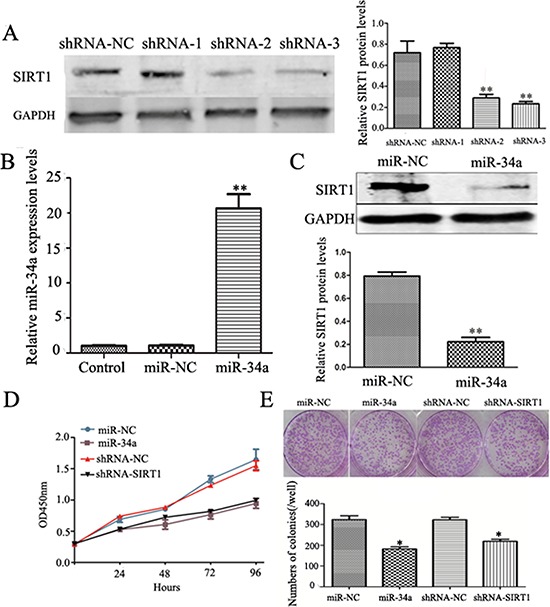
Down-regulation of SIRT1 and over-expression of miR-34a inhibit cell growth and colony formation abilities **A.** Comparison of shRNA-1, shRNA-2, and shRNA-3 in silencing SIRT1 expression at protein levels. MCF-7 cells were transfected with three shRNAs (shRNA-SIRT1–1, shRNA-SIRT1–2, and shRNA-SIRT1–3) respectively. Scrambling nucleotide sequence of SIRT1 (shRNA-NC) was used as negative control. After 48 h, cells were cultured under antibiotic pressure for 21 days and collected for Western blotting analysis (left). The quantitative results (right) were analyzed by Gel-Pro Analyzer 4.0 software, GAPDH was used as an endogenous control. **p < 0.01. **B.** MiR-34a mimics up-regulates miR-34a expression in MCF-7 cells. MCF-7 cells were transfected with miR-34a mimics (miR-34a) or non-specific control (miR-NC) for 48 h, and then collected for qRT-PCR analysis. **C.** Over-expression of miR-34a down regulates SIRT1 expression. Upper: Western blotting analysis the expression of SIRT1 in miR-34a or miR-NC transfected cells. Lower: The quantitative results were analyzed by Gel-Pro Analyzer 4.0 software, GAPDH was used as an endogenous control. **p < 0.01. **D.** CCK8 assay showed either ShRNA-SIRT1 or over-expression miR-34a inhibits the proliferation rate of MCF-7 cells. **E.** Either ShRNA-SIRT1 or over-expression miR-34a reduces the number and average size of colony in MCF-7 cells. Each condition was repeated 3 times and error bars represent SEM.

Our results above showed a reversible relationship between miR-34a and SIRT1 in BCSCs. Therefore, we speculated that manipulation of miR-34a-SIRT1 axis may interfere with the oncogenic properties of breast cancer cells. To test this hypothesis, we manipulated this axis by either silencing SIRT1 or ectopic expression of miR-34a in MCF-7 cells. Figure 2A showed that silenced SIRT1 gene by using shRNA-SIRT1 suppressed its protein expression significantly. Ectopic expression of miR-34a was successfully performed by transfected MCF-7 with miR-34a mimics, which caused overexpression of the transient miR-34a (Figure 2B). As expected, forced expression of miR-34a down-regulated protein expression of SIRT1 significantly (Figure 2C).

To further show the manipulated effects of the miR-34a-SIRT1 axis on proliferation of breast cancer cells, CCK-8 and colony formation assays were tested. As shown in Figure 2D, either up-regulated miR-34a or down-regulated SIRT1 inhibited cell proliferation significantly (Figure 2D). Colonies containing at least 50 cells were counted on day 10 after plating. The capability of colony formation in MCF-7 cells treated with either miR-34a or shRNA-SIRT1 was examined. The results showed that either enforced expression of miR-34a or silenced SIRT1 suppressed the colony forming capacity remarkably as compared to controls (Figure 2E). These results above revealed that manipulated expression of miR-34a-SIRT1 axis inhibited the cell proliferation and colony forming capacity of breast cancer cells.

### Repression of miR-34a-SIRT1 axis on the proportion of CD44+/CD24− BCSCs and mammosphere formation capacity

**Figure 3:**
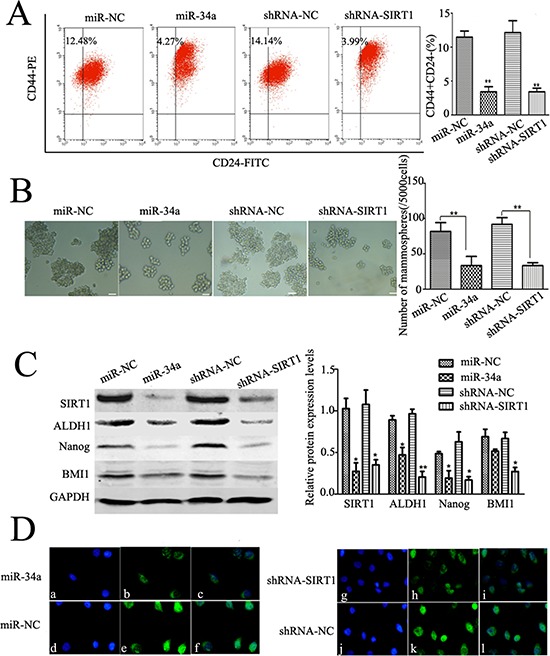
Dysregulation of miR-34a-SIRT1 axis decrease BCSCs **A.** The percentages of CD44^+^/CD24^−^ BCSCs were reduced either by over-expression of miR-34a or silencing SIRT1. The phenotype of CD44^+^/CD24^−^ cells was measured by flow cytometry. Similar results were obtained in three independent experiments, **p < 0.01. **B.** Either over-expression of miR-34a or down-regulation of SIRT1 decreased volume (*left*) and number (*right*) of mammospheres. After transfection with miR-34a mimics or shRNA-SIRT1, cells were cultured in ultra-low attachment plates with serum-free medium for 7 d. Mammospheres were collected and evaluated. **C.** SIRT1, ALDH1, Nanog, and BMI1 were down-regulated as over-expression of miR-34a or silencing SIRT1 (*left*). The quantitative results were analyzed by Gel-Pro Analyzer 4.0 software (*right*), and GAPDH was used as an endogenous control. *p < 0.05, **p < 0.01. **D.** Immunofluorescent staining confirmed lower Nanog protein expressions in miR-34a over-expressed cells and silenced SIRT1 cells. a, d, g, j, labeled with DAPI (a nuclear marker) (blue), b, e, h, k, FITC-labeled anti-Nanog (green), and c, f, i, l, represent overlay image of a and b, d and e, g and h, j and k, respectively. Scale bar = 50 μm.

To understand the regulatory mechanism of miR-34a-SIRT1 axis in BCSCs, cells were transfected with miR-34a mimics and shRNA-SIRT1. We found that the proportion sorted with FACS of CD44^+^/CD24^−^ BCSCs was significantly decreased in cells treated with either miR-34a (reduced by 65.85 ± 8.17%) or silenced SIRT1 (reduced by 68.93 ± 7.21%) as compared to controls (Figure 3A). These data suggest that suppressed SIRT1 activity by miR-34a (so-called miR-34a-SIRT1 regulatory axis) could reduce the population of the CD44^+^/CD24^−^ BCSCs.

Mammosphere formation is a typical property of CSCs that reflects the self-renewal potential of stem cells. To study the regulatory effects of miR-34a-SIRT1 axis on self-renewal of BCSCs, mammosphere formation assay was performed. We found that relaxed and smaller spheres were formed in the cultured BCSCs treated with either overexpression miR-34a or slienced SIRT1 as compared to the control cells (Figure 3B). The number of mammosphere was reduced by 48.93 ± 7.48% in cells treated with miR-34a, and by 57.78 ± 6.59% in cells treated with ShRNA-SIRT1 (Figure 3B). To verify the inhibitory effects, the capability of the secondary mammosphere formation was studied and showed that the secondary mammosphere formation capacity was reduced by 59.30 ± 3.57% or 65.03 ± 5.51% in cells treated with either miR-34a or ShRNA-SIRT1, respectively.

To further study the inhibitory effects of miR-34a-SIRT1 axis on BCSCs, expression of CSCs markers were studied, including BCSCs marker ALDH1, the self-renewal gene BMI1, and stem cell marker Nanog. Western blotting analysis revealed that protein expression of SIRT1, ALDH1 and Nanog was significantly decreased in BCSCs treated with either miR-34a or shRNA-SIRT1 (Figure 3C). Silenced SIRT1 suppressed BMI1 expression significantly compared to control, while BMI1 expression was down-regulated by miR-34a treatment even though there was no significantly difference as compared to control (Figure 3C). As demonstrated above, immunofluorescence of Nanog was decreased significantly in cells treated with either miR-34a or shRNA-SIRT1 compared to the control (Figure 3D). These data suggest that modulation of miR-34a-SIRT1 axis in BCSCs reduced the stemness of BCSCs *in vitro*.

### Induction of cell apoptosis by miR-34a-SIRT1 axis in MCF-7 cells

**Figure 4:**
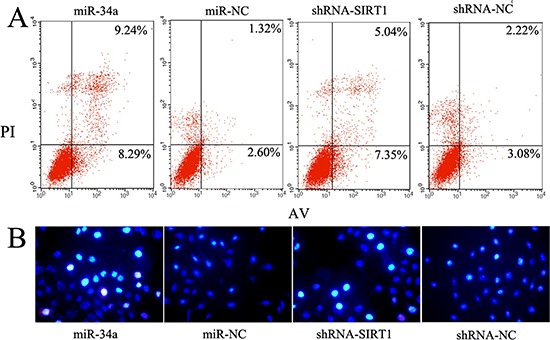
Modulation of miR-34a- SIRT1 axis enhanced MCF-7 cell apoptosis **A.** Over-expression of miR-34a and silenced SIRT1 increased the percentage of early period apoptotic cells labeled with Annexin V-FITC/PI. Representative scatter grams from flow cytometry profile represent Annexin V-FITC staining in x axis and PI in y axis. **B.** The apoptotic cells labeled with Hoechst 33342 increased either in miR-34a over-expression cells or silenced SIRT1 cells. The apoptotic cells emit strong blue fluorescence.

Self-renewal is an essential feature of stem cells, and escape from apoptosis can enhance the self-renewal capability of CSCs. We hypothesized that either up-regulated miR-34a or down-regulated SIRT1, reduced the self-renewal of BCSCs leading to cell apoptosis. To test this hypothesis, MCF-7 cells were transfected with either miR-34a (mimics) or shRNA-SIRT1, and further labeled doubly with Annexin V-FITC/PI. Cell apoptosis was determined by flow cytometry. A higher ratio of apoptotic and dead cells was observed in cells treated with either miR-34a or shRNA-SIRT1 compared to controls (Figure 4A). Meanwhile, Hoechst 33342/PI staining showed the nuclei of apoptotic cells emitting a shrunken, fragmented and strong blue fluorescence. Hoechst 33342 labels were increased by treated with either miR-34a or shRNA-SIRT1 as compared to control cells (Figure 4B). These results suggested that dysregulation of miR-34a-SIRT1 axis reduced the stemness and increased apoptosis of BCSCs.

### Inhibition effect of miR-34a-SIRT1 axis in breast tumor growth *in vivo*

**Figure 5:**
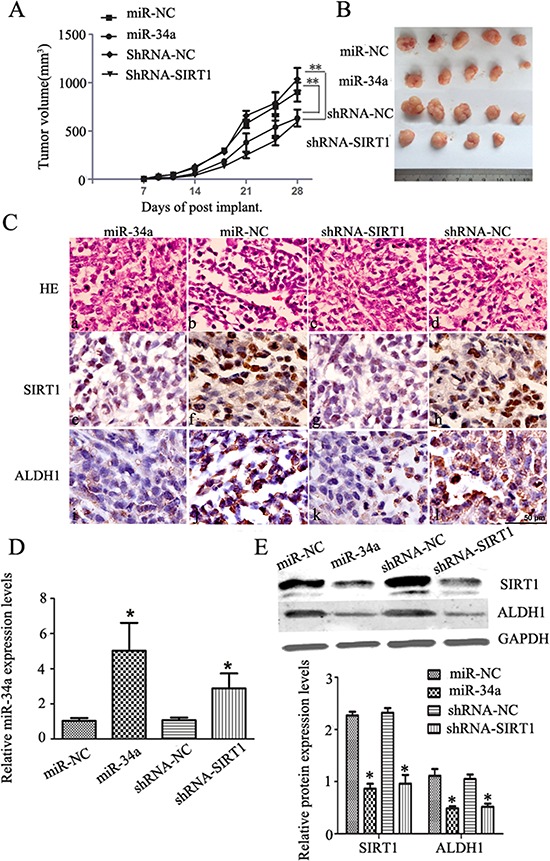
MiR-34a over-expression or silenced SIRT1 inhibit tumor growth *in vivo* **A.** Over-expression of miR-34a or silenced SIRT1 remarkably reduced the tumor volume compared to the control groups. *p < 0.05, **p < 0.01. **B.** Subcutaneous tumor regeneration from MCF-7 cells infected with lentivirus-miR-NC (miR-NC) or lentivirus-miR-34a (miR-34a), or transfected with shRNA-NC(shRNA-NC) or shRNA-SIRT1(shRNA-SIRT1). **C.** HE and IHC staining with SIRT1 and ALDH1 antibodies. a-d: HE staining; c-l: IHC staining for SIRT1 (brown color in nuclei) and ALDH1 (brown color in cytoplasm), scale bar = 50 μm. **D.** qRT-PCR analyses of miR-34a expression in each group of xenograft tissues. Compared to each control group, miR-34a or shRNA-SIRT1 group showed a significantly high miR-34a expression level. *p < 0.05. **E.** Protein levels of SIRT1, and ALDH1 were evaluated by Western blotting analysis in each group of mice tumors (*upper*). As an internal control, GAPDH was used for normalization. The quantitative results were analyzed by Gel-Pro Analyzer 4.0 software (*lower*). Data are presented as mean ± SEM, *n* = 5, *p < 0.05.

To further determine whether modulation of miR-34a-SIRT1 axis can inhibit tumorigenesis and tumor growth *in vivo*, we inoculated 5 × 10^5^ cells stably transfected with either lentivirus-miR-34a or shRNA-SIRT1, into mouse mammary fat pad. Four weeks post-inoculation, mice were sacrificed. We found that treatment of either overexpression miR-34a or silenced SIRT1 inhibited the tumor growth significantly as shown in Figure 5A. The macroscopic tumors were observed in 5 of 5 nude mice among groups of miR-34a and two control groups (miR-NC and ShRNA-NC) except for ShRNA-SIRT1 group, which the macroscopic tumors were observed only in 4 of 5 nude mice. The final tumor size in mice from groups treated either with shRNA-SIRT1 or miR-34a was significantly smaller than the control groups on day 28 when sacrificed (Figure 5B).

To further understand the underlying mechanism, expression of BCSCs marker in tumors from mice was studied by immunohistochemical analysis. Hematoxylin and eosin (H&E) staining analysis showed more extensive necrosis in mice treated with either miR-34a or shRNA-SIRT1 compared to controls (Figure 5C). Expression of SIRT1 and ALDH1 in mice treated with either miR-34a or shRNA-SIRT1 was down-regulated significantly as compared to the control mice (Figure 5C). To quantify the changes of miR-34a regulation in tumor tissues, qRT-PCR on miR-34a expression was performed. As expected, miR-34a expression was increased significantly in mice treated with either miR-34a or shRNA-SIRT1 compared to controls (Figure 5D). To understand the regulatory effects of miR-34a on BCSCs stemness, the expression levels of SIRT1 and ALDH1 in tumor tissues were measured by Western blot. Both SIRT1 and ALDH1 expression was significantly suppressed by either silenced SIRT1 or overexpressed miR-34a in tumor tissues (Figure 5E). Together, these data suggest that regulation of miR-34a-SIRT1 axis in BCSCs inhibited tumorigenesis and decreased ALDH1-positive cell population resulting in suppression of tumor growth in xenograft mice.

## DISCUSSION

Growing evidence shows that hidden CSCs contribute to drug resistance and tumor relapse [45, 46]. Therefore, CSCs as a novel strategy in cancer therapy have gained more interests. In the present study, we investigated the role of miR-34a and its target gene SIRT1 in the self-renewal of BCSCs *in vitro* and *in vivo*.

MiR-34a is one of the wildly studied cancer-associated microRNAs in the last few years [17, 22, 24, 25, 30, 47]. MiR-34a functions as a tumor suppressor and its down-regulation has been found in many malignant tumors including breast cancer, colon cancer, pancreatic cancer, neuroblastoma, hepatocellular carcinoma, and non-small cell lung cancer [23, 44, 48–50]. MiR-34a has been developed as a clinical predictor for evaluation of therapeutic outcomes of breast cancer. Higher miR-34a expression predicts lower risk of recurrence or death in breast cancer [51]. It was also reported that miR-34a inhibits proliferation, migration, and invasion of breast cancer cells by targeting several genes including E2F3, CD44, and SIRT1 [21], Notch1 and DLL1 [23]. Our current results confirmed the inhibitory effects of miR-34a overexpression and SIRT1 knock-down in the proliferative potential of breast cancer cell line.

MiR-34a is a downstream effector of p53 in normal and cancer cells [52]. It is directly transactivated by p53, and regulates a various genes that involving in cell-cycle progression, apoptosis, DNA repair, and angiogenesis [24]. SIRT1 is a known direct target of miR-34a. SIRT1 regulates p53-mediated apoptosis through deacetylating p53 [41]. Based on this, Yamakuchi et al. [41] proposed a positive feedback loop in miR-34a, SIRT1 and p53. In the present study, we observed that dysregulation of miR-34a-SIRT1 axis promoted apoptosis in MCF-7 cells. Therefore, in wild-type p53 breast cancer cell line, miR-34a may promote apoptosis through targeting SIRT1, which permits acetylation and activation of p53.

MiR-34a is also one of the well-studied miRNAs in various CSCs including prostate [28], pancreas [31, 32], and glioma [53]. MiR-34a inhibits self-renewal and metastasis of the prostate CSCs by repressing CD44 [28]. It represses the pancreatic CSCs by targeting bcl-2 and Notch [31]. MiR-34a could induce glioma stem cell differentiation [51]. Our previous study showed that down-regulation of Notch1 may help to eliminate BCSCs [45]. The role of SIRT1 in cancer has long been studied and debated [54–57]. As an oncogene, SIRT1 is over-expressed in the malignant tumors of prostate, breast, pancreas, and liver [29, 32, 33, 58]. SIRT1 also enhances pluripotent stem cells (iPSC) generation [59], and is expressed highly in hESC [60]. Inhibition of SIRT1 activates apoptosis of CD133^+^ glioma cells, and CML LSCs [40, 61]. Our results showed lower endogenous expression of miR-34a, whereas higher expression of SIRT1 in BCSCs. Either overexpression miR-34a or silenced SIRT1 resulted in lower proportion of BCSCs and decreased mammosphere formation capacity in MCF-7 cells. Expressions of ALDH1, BMI1 and Nanog were down-regulated in miR-34a or siRNA of SIRT1 transfecting cells. Moreover, our *in vivo* study indicated that either miR-34a overexpressing or SIRT1 knocking down MCF-7 cells significantly inhibited the growth of tumors. Meanwhile, ALDH1 expression was also suppressed. Thus, miR-34a plays a role in the self-renewal and maintenance of BCSCs possibly through targeting SIRT1.

In mESC, inhibition of SIRT1 leads to acetylation of p53, which further binds to the promoter of the Nanog to inhibit its expression, causes differentiation [39, 49, 44]. In our study, Nanog expression was increased in MCF-7 cells treated with miR-34a or siRNA of SIRT1. We speculated that miR-34a inhibits the self-renewal of BCSCs through targeting SIRT1, and subsequent inhibition of p53-dependent Nanog expression.

Taken together, our study demonstrated that over-expressed miR-34a decreased the proportion of BCSCs and inhibited its proliferation, showed a lower expression level of some CSCs related markers. Down-regulation of its direct target SIRT1 presented a similar effect on BCSCs. As a small molecular agent, miR-34a may help to eradicate BCSCs through down-regulating SIRT1 expression, induce a p53 dependent apoptosis. These results suggest that miR-34a-SIRT1 axis may play an essential role in the self-renewal and maintenance of BCSCs, which may help to devise BCSCs specific therapeutic strategies to improve cancer treatment.

## MATERIALS AND METHODS

### Ethics statement

Investigation has been conducted in accordance with the ethical standards and according to the Declaration of Helsinki and according to national and international guidelines and has been approved by the authors' institutional review board.

### Cell culture, reagents and animals

The human breast cancer cell line MCF-7 was obtained from Shanghai Culture Collection (Shanghai, China), and cultured as described previously [45]. The MCF-7 cell line was confirmed to have the wild-type p53 gene status. Antibodies against SIRT1, BMI1, Nanog, and GAPDH were purchased from Santa Cruz biotechnology Inc (Santa Cruz, CA, USA). Rabbit anti-ALDH1 antibody was purchased from Abcam (Cambridge, UK). Cell counting kit-8 (CCK-8) was purchased from DOJINDO laboratories (Kumamoto, Japan). The mammospheres were cultured using Complete MammoCult^TM^ Medium (Human)(Stemcell Technologies Inc, Vancouver, Canada), according to the manufacturer's instructions. Twenty 6-to 8-week-old female nude mice were purchased from the Key Laboratory for SPF Animals of Liaoning Province. Animal cares were conducted according to the protocols and guidelines approved by the Animals Care and Use Committee of Dalian Medical University.

### Isolation of CD44^+^/CD24^−^ BCSCs by microbeads

Mammospheres were harvested and enzymatically dissociated into single-cell suspension. Cell suspension was centrifuged at 300 × g for 10 minutes, and the cell pellet was resuspended in 40 μl suspension buffer (~10^7^ total cells). The cells were then incubated with CD24 Microbead Kit and CD44 Microbeads (Miltenyi Biotec, Bergisch Gladbach, Germany) for 15 minutes in refrigerator (4°C), then washed and resuspended in 500 μl buffer, followed by magnetic separation. The CD44^+^/CD24^−^ cells were then collected as the BCSCs.

### Over-expression of miR-34a in MCF-7 cells

The oligonucleotides of miR-34a mimics (miR-34a) and a non-specific negative control (miR-NC) were synthesized and purified by Invitrogen (Shanghai, China). The miR-34a and miR-NC were transfected by using Lipofectamine 2000(Life Technology, CA, USA) at a final concentration of 50 nM in Opti-MEM I Reduced Serum Medium (Life Technology, USA) according to the manufacturer's instructions. The transfected cells were harvested for RNA isolation and protein extraction at 48 h and 72 h post transfection, respectively. The miR-34a was cloned into GV254 vector with *NheI/NheI*. Lentivirus-hsa-miR-34a (3639–1) expression vector and control lentivirus-hsa-NC were obtained from Genechem (Shanghai, China), and infected MCF-7 cells at MOI of 40. Stable infected cells were then selected with 0.5 ug/ml puromycin (Sigma-Aldrich, USA) for 7 days.

### ShRNA-SIRT1 construction and transfection

The short hairpin RNA (shRNA) expression plasmids, *pGPHI/GFP/Neo-SIRT1*, were obtained from GenePharma (Shanghai, China). Three RNAi expression vectors for human SIRT1 (shRNA-SIRT1, shRNA-SIRT1–2, and shRNA-SIRT1–3) were constructed. Scrambled nucleotide sequence was used as a negative control (shRNA-NC), and shRNA-GAPDH was used as a positive control. These vectors were transfected into MCF-7 cells at a final concentration of 50 nM by using Lipofectamine 2000 according to the manufacturers' instructions. Transfection rates were confirmed by fluorescence microscopy. The positive transfected cells were further selected by using G418 at 350 mmol/L (Gibco Laboratories, Gran Island, NY) for 21 days.

### RNA extraction and quantitative real time RT-PCR (qRT-PCR)

Total RNA were extracted with TRIzol Reagent (Life Technology, USA) according to the manufacturers' instructions. MiR-34a reverse transcription was performed with the TaqMan microRNA Reverse Transcription Kit (Life Technology, CA, USA) using miR-34a specific RT primers. The qPCR amplification for miR-34a was performed with the TaqMan Universal PCR Master Mix II by using TaqMan Small RNA Assay according to the manufacturer's instructions (Life Technology, USA). RNU6B was used as an endogenous control. To determine mRNA levels of SIRT1 and GAPDH, cDNA was synthesized from 1 μg total RNA in 20 ul using a PrimeScript RT reagent kit (TaKaRa, Dalian, China). SYBR Premix Ex Taq II (TaKaRa, Dalian, China) was used for qPCR. Primers were used as follows,

SIRT1-F: 5′-CCCAGAACATAGACACGCTGGA-3′
SIRT1-R: 5′-ATCAGCTGGGCACCTAGGACA-3′
GAPDH-F: 5′-GCACCGTCAAGGCTGAGAAC-3′
GAPDH-R: 5′-TGGTGAAGACGCCAGTGGA-3′
GAPDH was used as an internal control. All samples were normalized to internal control, and fold changes were calculated through relative quantification (RQ = 2^−ΔΔCT^). All reactions were done in triplicate.

### Protein extraction and Western blot

Cells were collected and lysed in 1 × RIPA buffer (Sigma, St. Louis, MO). 50 μg of each protein extract was subjected to 8~12% SDS-PAGE gels and transferred to PVDF membrane (Millipore, Billerica, MA). The resulted blots were first probed with the primary antibodies, including SIRT1 (1:300), ALDH1 (1:500), BMI1 (1:200), and Nanog (1:200), and then probed with infrared dye reagents IRDye 800 CW (1:5000) as the secondary antibody.

### Immunofluorescence

Cells were fixed with 4% paraformaldehyde, blocked with 10% normal goat serum (Gibco Laboratories, Gran Island, NY), and incubated with anti-SIRT1 (1:100) or anti-Nanog (1:100) overnight at 4°C. Immunolabeling was revealed by FITC or PE-conjugated secondary antibodies. Nuclei were counterstained with DAPI. Immunofluorescence was observed under a Nikon Eclipse 300 fluorescence microscope (Compix Inc, Sewickley, PA, USA).

### CCK-8 assay

Cells were plated in 96-well plates at a density of 2 × 10^3^ per well. At 0 h, 24 h, 48 h, 72 h, and 96 h post-plating, 10 μl Cell Counting Assay Kit-8 solution was added to each well and incubated for 2 h, the absorbance at 450 nm was measured by a microplate reader. Addition of DMEM/F12 alone was used as black control. All experiments were performed three times independently.

### Colony formation assay

Cells were trypsinized, counted, and seeded for colony formation assay in 6-well plates at 1000 cells per well. During colony growth, the culture medium was replaced every 2 days. The colony was counted only if it contains more than 50 cells, and the number of colonies was counted at 7 days after seeding. Each treatment was carried out in triplicate.

### Cell apoptosis analysis

Cells were harvested and resuspended in 500 μl of binding buffer and stained with 5 μl of FITC-Annexin-V (BD Biosciences, San Jose, CA) and 5 μl of propidium iodide (Sigma, St. Louis, MO) for 30 min in the dark at 4°C. Cells were analyzed by flow cytometry. This experiment was repeated three times.

### Staining with Hoechst 33342/PI

Cells were washed twice in PBS and fixed in PBS containing 1% (wt/vol) paraformaldehyde (Fisher Scientific, Pittsburgh, PA). After rinsing with water, cells were stained with Hoechst. 33342/PI (Sigma, St. Louis, MO) for 30 min. The morphologic aspect of nuclei was observed under a fluorescence microscope using a 320 to 350 nm filter. This experiment was repeated three times.

### Flow cytometry analysis of CD44^+^/CD24^−^ cell populations

Cells were collected and labeled with PE mouse anti-human CD44 (Biolegend, USA) and FITC mouse anti-human CD24 (Biolegend, USA) at a density of 10^6^ per ml. After 30 min incubation at 4°C, staining cells were sorted using BD FACS Calibur Flow Cytometer (BD, Franklin Lakes, NJ).

### Mammosphere formation assay

Different groups of cells were inoculated at a density of 4 × 10^4^ cells/well in ultra-low attachment 6-well plates (Corning, NY, USA) and grown in Complete MammoCult^TM^ Medium (Human). After 7 days culture, colonies that larger than 60 μm in size were counted. In order to evaluate the self-renewal ability, mammospheres were dissociated with pre-warmed Trypsin-EDTA and single-cell suspension was seeded. The secondary sphere formation was measured as described above.

### Tumor formation, growth and morphologic analysis *in vivo*

Twenty nude mice were randomly divided into four groups, group 1 were injected with lentivirus-hsa-NC (miR-NC) infected cells, group 2 were injected with the lentivirus-hsa-miR-34a (miR-34a) infected cells, group 3 were injected with shRNA-Control (shRNA-NC) transfected cells, and group 4 were injected with shRNA-SIRT1 transfected cells. 5 × 10^5^ cells in each group were resuspended in 100 μl PBS mixed with matrigel (1:1) and injected into the mouse mammary fat pad. The tumor mass was monitored using a caliper. Tumor volumes (V) were calculated by the formula *V* = *L* × *W*^2^ × 0.5. On day 28 postinoculation, mice were sacrificed. The transplanted tumors were excised and frozen in liquid nitrogen until processing for isolation of RNA and protein. For histological study, portion of tumors were fixed in 10% neutral-buffered formalin, paraffin-embedded and 4-μm sections were stained for H&E. For immunohistologic assay, SIRT1 and ALDH1 were detected by anti-SIRT1 and anti-ALDH1 primary antibodies, respectively.

### Statistical analysis

All experiments were performed independently in triplicate. The results are presented as the mean μ ± SEM. Statistical analysis was performed using a two-way Student's *t*-test. Statistical significance was set as a *p < 0.05, **p < 0.01, respectively.
